# Surgical Removal of Cranial Calcium Phosphate Bone Paste Micro-fragmented by Postoperative Head Trauma: A Case Report

**DOI:** 10.7759/cureus.88934

**Published:** 2025-07-28

**Authors:** Issei Asari, Ayumu Yamaoka, Yukinori Akiyama, Yuuki Sakurai, Nobuhiro Mikuni

**Affiliations:** 1 Department of Neurosurgery, Sapporo Medical University, School of Medicine, Sapporo, JPN; 2 Department of Radiology, Sapporo Medical University Hospital, Sapporo, JPN

**Keywords:** calcium phosphate bone paste, high-energy trauma, micro-fragmentation, motor vehicle accident, removal surgery

## Abstract

Calcium phosphate bone paste (CPBP) is widely used in cranioplasty, yet its strength under extreme stress and optimal management of subsequent post-traumatic fragmentation remain poorly defined. The aim of this paper is to illustrate the limitations of CPBP durability through a case in which even seemingly hardened material underwent micro-fragmentation due to high-energy trauma after cranioplasty, and to explore the necessity of surgical intervention in such cases. We present a case of a woman in her 30s with multiple complex comorbidities, including immunodeficiency requiring steroid therapy, a fibrinogenolytic system disorder, and fluid management difficulties necessitating diuretics due to protein-losing enteropathy. She underwent cranioplasty with CPBP following resection of a ruptured cerebral arteriovenous malformation (AVM). Two months post-AVM resection, a motor vehicle accident (MVA) caused significant damage to the cranioplasty site, including displacement of the titanium plate, migration of the bone flap, and micro-fragmentation of the CPBP. Initially managed conservatively due to her critical condition, the patient subsequently developed localized pain, erythema, and alarming skin thinning at the micro-fragmentation sites. Given the high risk of impending ulceration and infection, particularly in a patient with underlying medical complexities, surgical removal of the micro-fragmented CPBP and associated hardware was performed five months post-MVA. The procedure successfully alleviated her symptoms without further complications. This case illustrates that CPBP, despite its benefits, may not withstand high-impact forces such as those sustained in an MVA, leading to micro-fragmentation. Crucially, it underscores that even seemingly minor fragmentation, if associated with progressive local symptoms or complex patient factors, may necessitate proactive surgical intervention to prevent severe complications like ulceration and infection.

## Introduction

Calcium phosphate bone paste (CPBP) has emerged as a widely utilized material for cranial bone defect reconstruction due to its remarkable intraoperative moldability, excellent biocompatibility, and reportedly lower rates of wound infection and cerebrospinal fluid leakage compared with titanium mesh [1-3]. However, with the widespread adoption of CPBP, complications such as foreign body reaction [4-6], extrusion [7,8], and infection [4,6-10] have been reported. In particular, with respect to postoperative fracture, concerns persist regarding the inherent mechanical strength of CPBP and its susceptibility to fracture, with several such instances having already been documented [4,7-11]. While previous case series often describe CPBP failures associated with relatively large bone defects leading to severe complications such as ulceration or overt infection [4,5,8,10], there remains significant clinical ambiguity regarding the optimal indications and precise timing for surgical removal of CPBP that has undergone micro-fragmentation without immediate signs of ulceration or gross infection. The aim of this paper is to illustrate the limitations of CPBP durability and to explore the necessity of surgical intervention in such cases. This report addresses this critical gap by presenting a unique and instructive case of CPBP micro-fragmentation stemming from direct blunt trauma to a craniotomy site, occurring just two months after surgery due to a motor vehicle accident (MVA). Our experience offers valuable insight into the challenges of managing such subtle yet progressive CPBP failures and emphasizes the potential need for early surgical intervention, even in the absence of overt complications.

## Case presentation

**Figure 1:**
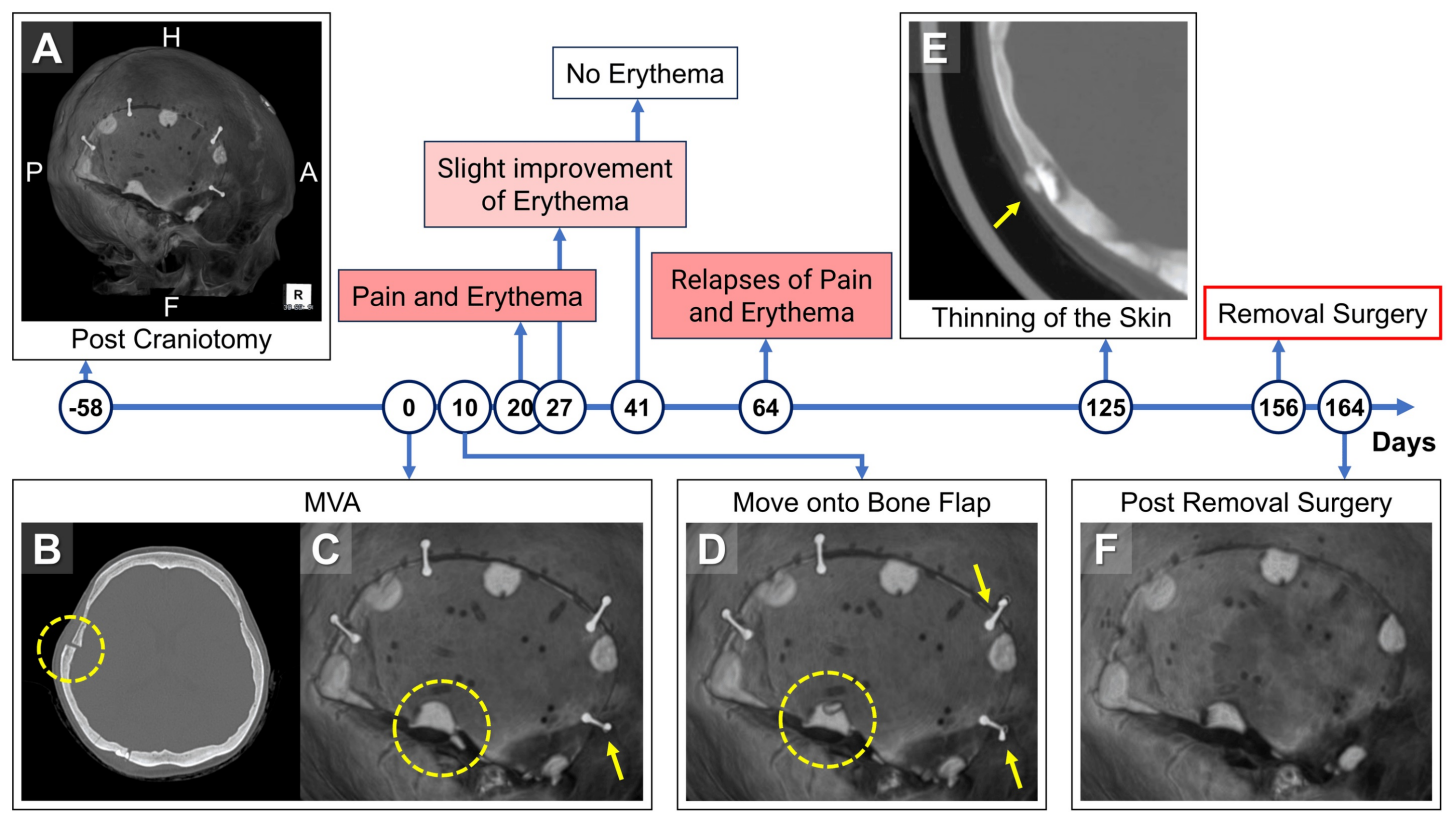
Comprehensive clinical and radiological course of this case (A) The bone flap was filled with calcium phosphate bone paste (CPBP) after resection of the cerebral arteriovenous malformation. (B, C) Following admission of the patient after a traffic accident, dislodgement of the titanium plate and screws, migration of the bone flap, and micro-fragmentation of CPBP filled with the right temporal base were apparent (circle). (D) The micro-fragments of CPBP moved onto the bone flap 10 days after admission (circle and arrows). (E) Thinning of the skin could be observed above the micro-fragmentation of CPBP four months after the accident. (F) Postoperative computed tomography after removal of micro-fragments of CPBP and the plates. MVA: motor vehicle accident

A woman in her 30s, with a complex medical history including immunodeficiency requiring chronic steroid therapy, a challenging fibrinogenolytic system disorder, and fluid management difficulties necessitating diuretics due to protein-losing enteropathy, underwent microsurgical resection for a ruptured cerebral arteriovenous malformation. This involved a right temporal craniotomy and mastoidectomy, with the bone defect meticulously filled using CPBP (Figure 1A). Her postoperative course was initially uneventful. However, due to the need for rehabilitation after the ruptured AVM and a minor complication at the puncture site related to endovascular treatment, she was discharged home after 28 postoperative days. However, just two months after surgery, her course took an unexpected turn. The patient was readmitted following a high-impact MVA, in which her craniotomy site sustained a direct hit while she was cycling. Head computed tomography (CT) immediately revealed traumatic subarachnoid hemorrhage and a contralateral frontal lobe cerebral contusion. More critically, imaging of the craniotomy site showed clear evidence of dislodgement of the titanium plate and screw, migration of the bone flap, and extensive micro-fragmentation of the CPBP within the right temporal base (Figures 1B-1C). Given the patient’s immediate post-MVA status, complicated by convulsive status epilepticus requiring deep sedation and tracheal intubation, initial management of the craniotomy site injuries was conservative. Ten days later, follow-up CT indicated some movement of the CPBP micro-fragments onto the bone flap (Figure 1D), yet the patient remained asymptomatic in this area. Her epilepsy was subsequently controlled, leading to her discharge 17 days after the MVA, with careful outpatient monitoring.

The clinical picture began to evolve three weeks post-MVA. The patient presented with localized pain and erythema in the skin overlying the CPBP micro-fragmentation site. Although no desquamation, ulceration, or overt signs of infection were present, her underlying comorbidities, coupled with the prior mastoidectomy and its complex repair, raised significant concerns about the potential for future infection if reoperation were to occur. Blood tests were performed multiple times but showed no signs of infection. White blood cell counts remained within the normal range (2,300-7,500/µL), and although CRP levels were mildly elevated (0.03-0.57 mg/dL), there was no upward trend. Consequently, conservative treatment continued. Despite some temporary improvement in erythema at four and six weeks, the protuberances persisted. Two months after the MVA, the patient again complained of recurrent pain and erythema in the area. A follow-up head CT four months post-MVA revealed significant thinning of the skin directly above the micro-fragmented CPBP (Figure 1E). This alarming finding, coupled with the persistent local symptoms and the inherent risks associated with her medical conditions, prompted a decisive shift in our management strategy. Five months after the MVA, we opted for surgical removal of the micro-fragmented CPBP to preempt the inevitable development of skin ulcers or deep-seated infections. Concurrently, damaged plates and screws were removed, and the dislocated bone flaps were realigned.

**Figure 2:**
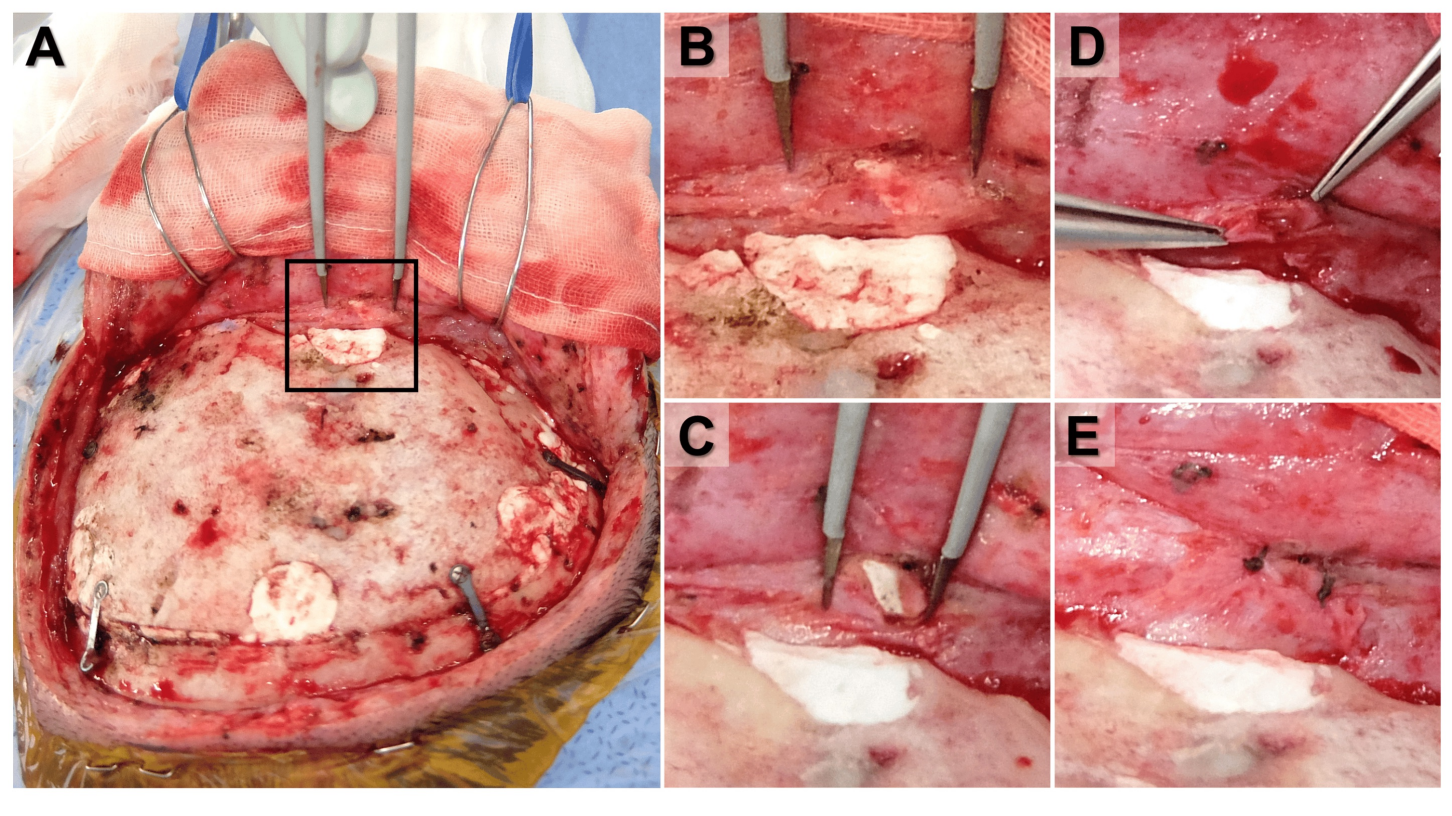
Photographic images of removal surgery of the micro-fragmented calcium phosphate bone paste (CPBP) (A) The previous incision line was utilized, and the skin flap was inverted from the layer under the periosteum; (B) the micro-fragmentation of CPBP was entrapped within the periosteum; (C) the membrane covering the micro-fragmentation of CPBP was incised; (D) the micro-fragmentation of CPBP was removed completely; and (E) the incision was sutured with absorbable sutures.

The surgery proceeded using the previous incision line, carefully inverting the skin flap from the subperiosteal layer. The bone flap was approximately fusion, and there was no evidence of infection (Figure 2A). We discovered CPBP micro-fragments entrapped within the periosteum, covered by a distinct membrane (Figure 2B). Upon incising this membrane, the fragments were found to be invaginated within loose surrounding tissue (Figures 2C-2D), necessitating complete removal (Figure 2E). The displaced bone flap margin was smoothly reshaped. Considering the ongoing burden on the skin, an absorbable plate was used for fixation to avoid future titanium-related skin issues. The postoperative CT confirmed the complete removal of the micro-fragmented CPBP (Figure 1F). Her postoperative course was excellent, with no recurrence of pain or skin erythema eight months after surgery, and no worsening of her underlying medical conditions.

## Discussion

This compelling case highlights two critical clinical considerations for neurosurgeons utilizing CPBP in cranioplasty: first, the potential for CPBP to sustain significant damage from high-energy trauma, even when the affected area is small; and second, the need to consider early surgical intervention for micro-fragmented CPBP based on evolving clinical and radiological findings, even in the absence of overt ulceration or infection.

Vulnerability of CPBP to high-energy trauma

While CPBP typically forms a cement-like structure within 12 to 52 weeks [3] and has been reported to withstand impact forces up to approximately 1200 N [12], its actual compressive strength exhibits wide variability (12-80 MPa), influenced by factors such as formulation, porosity, and curing conditions [13-15]. In our case, the patient’s CPBP had been in situ for only two months, suggesting it may not have reached its fully hardened state. Although precise numerical data for car-bicycle collision impact forces are complex and depend on numerous variables (speed, mass, duration, angle), a simple calculation for this patient (45 kg, hit at 40 km/h with an estimated impact time of 0.1 seconds) yields an approximate impact force of 5,000 N. This theoretical estimation significantly exceeds reported CPBP impact durability, strongly suggesting that CPBP may be inherently vulnerable to the forces encountered in high-energy MVAs, regardless of its hardening stage.

To prevent these mechanical complications, the indications for CPBP use must also be carefully evaluated. The appropriate defect size for which CPBP is suitable remains a subject of debate [1]. Conventionally, CPBP is deemed appropriate for larger cranial defects; however, serious complications have also been reported in such cases, particularly when trauma is involved [10,16]. Nevertheless, our case presents a striking paradox: it clearly demonstrates that even a small amount of CPBP can undergo traumatic micro-fragmentation, necessitating surgical intervention. In this case, CPBP was selected because the patient was a young woman with cosmetic concerns, such as scalp depression. Therefore, the decision to use CPBP should be based on careful consideration of the patient's individual background and clinical context. These findings underscore the importance of avoiding the unnecessary use of CPBP, as well as the critical need for increased caution and specific patient education regarding head trauma risks for individuals who have undergone cranioplasty with CPBP. Clinicians should be acutely aware that micro-fragmentation of CPBP can occur even with seemingly subtle head trauma and necessitates diligent follow-up.

Optimizing surgical intervention for micro-fragmented CPBP

The long-term behavior of CPBP fragments remains a topic of ongoing study. Although CPBP ossification is often limited to the periphery, with the majority of the cement not fully replaced by bone [17,18], and reported partial absorption rates averaging 25% [16], some evidence suggests that impaired blood flow or existing fractures may inhibit its resorption [1]. Moreover, studies on CPBP use for transpetrosal procedures have reported spontaneous ejection in a notable percentage of cases (7.3%), with a wide timeframe for ejection (within one year in 23.1% of cases and 20-140 months in 76.9% of cases) [8]. These findings indicate that relying solely on natural ejection can be a prolonged and uncertain process.

More critically, CPBP fragments are thought to induce chronic inflammation and foreign body reactions, potentially leading to progressive skin thinning, fistula formation, and wound dehiscence [4,11,12,19]. While overt infection (reported mean rate of 5%) [1] significantly exacerbates these mechanisms, particularly when CPBP is placed near the petrous bone [8] or frontal sinus [5], our case highlights a more insidious progression. Two months post-craniotomy, the skin and bone flaps had not yet achieved full adhesion, allowing the fractured CPBP to migrate onto the bone flap. This likely caused recurrent skin irritation and progressive thinning due to mechanical irritation from the sharp fragments.

Despite intraoperative findings of CPBP micro-fragments covered by a membrane, suggesting a potential for eventual spontaneous ejection, we determined that prolonged observation was not feasible given the patient’s complex medical background, specifically immunodeficiency requiring steroid therapy, and the proximity of the CPBP to the mastoid air cells. These factors heightened the risk of severe infection if skin integrity were further compromised. Therefore, we advocate for a proactive treatment strategy of surgically removing micro-fragmented CPBP when it migrates onto the bone flap, is accompanied by inflammatory skin lesions, is positioned near compromised anatomical spaces like mastoid air cells, or occurs in patients with multiple comorbidities. This approach prioritizes patient safety and aims to prevent future, potentially catastrophic, complications rather than awaiting uncertain natural ejection.

## Conclusions

This report definitively demonstrates that head trauma following cranioplasty with CPBP can result in severe micro-fragmentation, even under circumstances where the material is expected to be stable. Our case underscores the absolute necessity of rigorous and prolonged follow-up for patients with micro-fragmented CPBP, even if initial symptoms are subtle. Furthermore, we strongly recommend that surgical intervention for CPBP micro-fragmentation be proactively considered based on the constellation of evolving clinical symptoms (e.g., progressive pain, erythema, skin thinning), specific radiological findings (e.g., fragment migration, dermal compromise), and, critically, the patient’s underlying medical complexities. This proactive approach can significantly mitigate the risk of severe future complications and improve long-term patient outcomes.
